# Cavernous Sinus Thrombosis: Efficiently Recognizing and Treating a Life-Threatening Condition

**DOI:** 10.7759/cureus.17339

**Published:** 2021-08-20

**Authors:** Selma Ali

**Affiliations:** 1 Department of Family Medicine, Premier Family Medicine, Denver, USA; 2 Department of Emergency Medicine, National Ribat University, Khartoum, SDN

**Keywords:** cavernous sinus thrombosis, septic thrombosis, dural sinuses, atypical cerebral venous thrombosis, acute bacterial sinusitis

## Abstract

Cavernous sinus thrombosis (CST) can develop as a result of both infectious and noninfectious conditions. Infections in the middle part of the face caused by Staphylococcus aureus are the most common cause of septic thrombosis of the cavernous sinuses. Paranasal (typically sphenoid) sinusitis, dental abscess, and, less commonly, otitis media are other antecedent sources of infection. Fever is almost always present, but a headache may not be noticeable. In almost all cases, periorbital edema, chemosis, proptosis, and a restriction of extraocular movements (particularly lateral gazing) emerge. Within two days of the development of unilateral symptoms, involvement of the opposite eye is common. Although CT can be useful, MRI is likely to be the preferred diagnostic method. Antibiotics and occasionally surgical drainage of the infection's primary focus are used to treat it. Complete recovery is about 50% while the death rate is around 30%.

## Introduction

Cavernous sinus thrombosis (CST) is a rare, life-threatening condition that can occur as a result of a face infection, sinusitis, orbital cellulitis, pharyngitis, or otitis media. It can also result from a serious injury or surgery, especially in the presence of a thrombophilic disorder. Fever, headache, periorbital edema, and ophthalmoplegia are some of the more common symptoms of CST, and early identification is critical for a favorable outcome. Despite the use of modern antibiotics and anticoagulants, the risk of long-term consequences such as vision loss, diplopia, and stroke continue to be substantial [1]. Isolated intracranial pressure syndrome, localized neurological impairments, and cavernous sinus syndrome are the most common clinical manifestations of this complex disease. The gold standard test modality is an MRI with a magnetic resonance venogram (MRV) [2]. Because CST is so infrequent, statistics on its occurrence are scarce. The annual incidence of CST can be estimated to be as high as two to four per million people per year [1] with an even higher incidence in children.

## Case presentation

The patient was a male of African descent, in his mid-60s who worked as a farmer. Medical history was notable for chronic sinusitis, seasonal allergies, atopic dermatitis, and type I diabetes mellitus on sulfonylurea otherwise, he maintained a healthy lifestyle. Normal BMI and surgical history were noted. Past medical history was significant for diabetic ketoacidosis (DKA) admission. The patient had been suffering from an intractable headache for two days that had not responded to acetaminophen, non-steroidal anti-inflammatory drugs (NSAIDs), or homeopathic medicines. Graded as severe and non-radiating, there were no aggravating or relieving factors. There are no other associated symptoms. The systemic review revealed an episode of sinusitis that commenced six days prior. On physical examination, vital readings were within normal limits and the patient did not show any signs of pallor or jaundice. He was oriented to time, place, and person and spoke in a comprehensive manner. He also maintained eye contact, a normal posture, and was not bothered by ER noise or lighting. Eye examination revealed isolated bilateral lateral gaze palsy. It was difficult to assess redness around the orbital area due to skin tone, but there was no orbital or periorbital edema, chemosis, proptosis, or any retinal findings. His vision examination revealed normal visual acuity and pupillary reflexes. There was mild tenderness over the maxillary and frontal sinuses, but otherwise a normal systemic examination. There were no signs of meningeal irritation or sepsis/septic shock.

Compilation of the presenting symptoms gives way to a number of differential diagnoses including but not limited to CST, acute angle-closure glaucoma, subdural hematoma, subarachnoid hematoma, epidural and/or orbital infections. CST was my most probable diagnosis due to the history of chronic sinusitis and diabetes mellitus. It was the most concerning differential diagnosis since it has the highest probability of mortality, as well as the highest rate of complications or dreadful outcomes of any of the other differentials. Preliminary blood test results showed slight leucocytosis with a slight shift to the left. Glycated hemoglobin was at 10%. Blood cultures, lumbar puncture (LP), and cerebrospinal fluid (CSF) examinations were ordered but not carried out once again due to socio-financial reasons. It is highly unlikely the maxillary sinuses were the cause behind the CST, as the most associated with complications are the ethmoid and sphenoid sinuses due to their proximity to the cavernous sinus. Given this somewhat vague yet alarming clinical picture, we decided to treat the patient for CST despite an incomplete diagnostic profile. It was agreed that the fatal outcome of CST outweighs the risks of antibiotic and anticoagulant administration.

For the treatment, our conflict revolved around two main points: first, the patient's limited capabilities (lack of health insurance/low income), and second, the use of antibiotics and blindly treating solely on our degree of suspicion. Blood cultures, LP, MRV, and other tests were not performed to our liking. After much deliberation, it was decided to start empirical antibiotics alongside low molecular weight heparin (LMWH) and corticosteroids. A non-contrast CT scan to rule out brain hemorrhage/hematoma was carried out prior to the administration of LMWH. The fact that the lateral gaze was bilateral rather than unilateral further contributed to our judgment that it was CST. A unilateral gaze could be attributable to a variety of circumstances, including but not limited to brain stem tumors or stroke, but a bilateral lateral gaze palsy almost always confirms cerebral venous thrombosis given the presenting picture and history. Antibiotics used were IV ceftriaxone, IV metronidazole, IV dexamethasone, and enoxaparin. The patient was then admitted to the ICU for additional close monitoring and management. Symptoms of complications such as meningitis, septic emboli, blindness, cranial nerve palsies, and sepsis/septic shock were all closely monitored. Even with the commencement of antibiotics, this period is exceedingly risky, with complication rates as high as 30%. Daily lab work and physical examinations were carried out on the patient, as well as supportive treatment. He maintained stable vital signs and did not develop any complications. He was kept on antibiotics and LMWH for another week before being transferred to the general ward. After making a full recovery, he was discharged home three weeks later and was scheduled for follow-up in the outpatient neurology department. The patient and his family were educated on the dangers of CST, its etiology, how to manage his diabetes and acute sinusitis episodes, how to recognize CST symptoms, and when to call his doctor or go to the ER.

## Discussion

Thrombosis of the cerebral venous system is an uncommon location of thrombosis, having a high frequency in young adults. Because of advancements in neuro-radiological procedures and an increased level of efficient diagnosis, this incidence has risen in recent decades [2-3]. Some risk factors for cerebral venous sinus thrombosis are shared with other venous thromboembolism sites, but others are unique to this anatomical area. Even if acute problems or chronic invalidity occur in a quarter of patients, the prognosis is generally positive if the diagnosis is recognized quickly and treatment is started swiftly. Anticoagulation is the cornerstone of treatment, as it is required to stop clot propagation and achieve re-canalization. Anticoagulation is not contraindicated in the presence of intracranial hemorrhage. Although data from clinical trials is insufficient, endovascular operations are reserved for patients with a particularly severe presentation or fast diminishing neurological symptoms despite proper anticoagulation [4-5].

CST is a medical emergency since it has such a devastating effect and can lead to major problems. Despite modern advancements, diagnosing CST is a difficult undertaking. Attempts to diagnose and treat patients as soon as possible necessitate a strong index of suspicion and a thorough understanding of the disease. Patients with CST, unfortunately, do not usually present with conventional symptoms, making identification all the more difficult [6-7].

**Figure 1:**
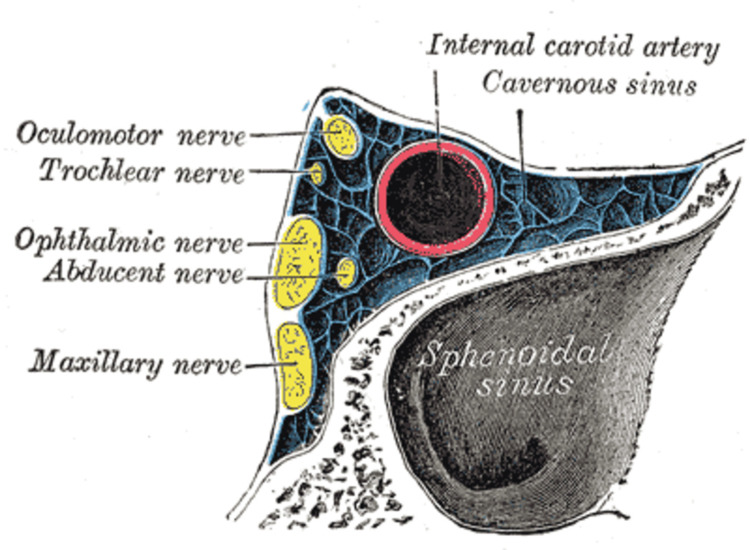
Anatomy of the cross-section of cavernous sinus showing close proximity to cranial nerves and sphenoid sinus. Henry Gray (1918) Anatomy of the Human Body. No permission is required to reproduce the image.

The cavernous sinuses are trabeculated cavernous cavities generated by the layers of dura mater and filled with venous blood (one on each side of the sella turcica, above and lateral to the sphenoid sinuses, anteriorly superior orbital fissure, and posteriorly petrous section of the temporal lobe). The superior and inferior ophthalmic veins, as well as the superficial cortical veins, drain into the basilar plexus anteriorly, whereas the superior and inferior petrosal sinuses drain into the basilar plexus posteriorly [8-10]. The close proximity of veins, arteries, nerves, meninges, and paranasal sinuses contributes to the development and presentation of CST as can be observed from Figure 1. The most prevalent causes of CST are sphenoid and ethmoid sinusitis, as well as frontal sinusitis. Over 70% of all infections are caused by the bacteria Staphylococcus aureus. There are also Streptococcus pneumoniae, Gram-negative bacilli, and anaerobes present. Fungi, which include Aspergillus and Rhizopus species, are a less common pathogen [9-10].

CST had a near 100% fatality rate before the introduction of powerful antibiotic treatments. The most prevalent causes of death are sepsis or a CNS infection. Because of rigorous management, the mortality rate has plummeted below 30%. However, morbidity is still significant, and full recovery is rare. These high rates of death and morbidity could be due to delayed diagnosis and antibiotic treatment. Although the average age is 22, it affects people of various ages. Visual impairment affects about one-half of people, whereas cranial nerve disorders affect the other half [10]. CT and MRI imaging are the most optimal diagnostic tests and can sometimes show early signs of clot formation which can greatly benefit management and case outcome.

## Conclusions

Patients with CST have a range of outcomes, from complete healing to chronic neurological damage. Approximately 10% had persistent neurological damage by the 12th month of follow-up. Anticoagulation in CST was a controversial and much-discussed topic as it could not be determined if the risks and complications outweigh the pros. Recent studies from previous clinical trials and observational research have demonstrated that anticoagulation is recommended and regarded as both safe and effective for treatment in CST. Patients with risk factors and ocular symptoms suggestive of CST should undergo early diagnostic imaging with contrast-enhanced CT or MRI. When surgical drainage is required, broad-spectrum intravenous antibiotics, anticoagulation, and early administration of broad-spectrum IV antibiotics are used. The take-home lesson from this article is that it is critical for ED physicians to recognize and act on mild indications of dural sinus thrombosis. The second point to mention is that in nations with low healthcare capacity, such as the one mentioned in this article, it may be difficult to obtain a thorough diagnostic profile, and a considerable reliance is placed on clinical presentation and clinician physical examination.
